# A Protocol for a Feasibility and Acceptability Study of a Participatory, Multi-Level, Dynamic Intervention in Urban Outreach Centers to Improve the Oral Health of Low-Income Chinese Americans

**DOI:** 10.3389/fpubh.2018.00029

**Published:** 2018-02-14

**Authors:** Mary E. Northridge, Sara S. Metcalf, Stella Yi, Qiuyi Zhang, Xiaoxi Gu, Chau Trinh-Shevrin

**Affiliations:** ^1^Department of Epidemiology and Health Promotion, College of Dentistry, New York University, New York, NY, United States; ^2^Department of Geography, The State University of New York at Buffalo, Buffalo, NY, United States; ^3^Department of Population Health, School of Medicine, New York University, New York, NY, United States

**Keywords:** implementation science, feasibility, acceptability, oral health, dental care, health equity, urban health, Chinese American

## Abstract

**Introduction:**

While the US health care system has the capability to provide amazing treatment of a wide array of conditions, this care is not uniformly available to all population groups. Oral health care is one of the dimensions of the US health care delivery system in which striking disparities exist. More than half of the population does not visit a dentist each year. Improving access to oral health care is a critical and necessary first step to improving oral health outcomes and reducing disparities. Fluoride has contributed profoundly to the improved dental health of populations worldwide and is needed regularly throughout the life course to protect teeth against dental caries. To ensure additional gains in oral health, fluoride toothpaste should be used routinely at all ages. Evidence-based guidelines for annual dental visits and brushing teeth with fluoride toothpaste form the basis of this implementation science project that is intended to bridge the care gap for underserved Asian American populations by improving access to quality oral health care and enhancing effective oral health promotion strategies. The ultimate goal of this study is to provide information for the design and implementation of a randomized controlled trial of a participatory, multi-level, partnered (i.e., with community stakeholders) intervention to improve the oral and general health of low-income Chinese American adults.

**Methods:**

This study will evaluate the feasibility and acceptability of implementing a partnered intervention using remote data entry into an electronic health record (EHR) to improve access to oral health care and promote oral health. The research staff will survey a sample of Chinese American patients (planned *n* = 90) screened at three outreach centers about their satisfaction with the partnered intervention. Providers (dentists and community health workers), research staff, administrators, site directors, and community advisory board members will participate in structured interviews about the partnered intervention. The remote EHR evaluation will include group adaptation sessions and workflow analyses *via* multiple recorded sessions with research staff, administrators, outreach site directors, and providers. The study will also model knowledge held by non-patient participants to evaluate and enhance the partnered intervention for use in future implementations.

## Introduction: Background and Rationale

**Figure 1:**
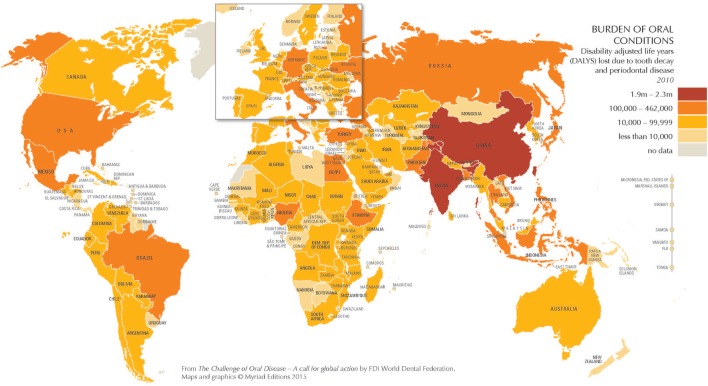
The burden of oral conditions worldwide as measured by disability adjusted life years lost due to tooth decay and periodontal disease, 2010. Source: Benzian and Williams (1). Printed with permission.

In 2010, oral health conditions affected 3.9 billion of 6.9 billion people worldwide, or over half (57%) of the global population (1). Indeed, untreated tooth decay (dental caries) was the single most prevalent and severe periodontitis (periodontal disease) was the sixth most prevalent of 291 oral and general health conditions studied (1). The burden of unmet oral health needs on quality of life is substantial, especially for populations with fewer resources. Globally, disability adjusted life years (DALYs) lost due to dental caries and periodontal disease is considerable, especially in China and India (Figure 1), but also for disadvantaged populations worldwide.

**Figure 2:**
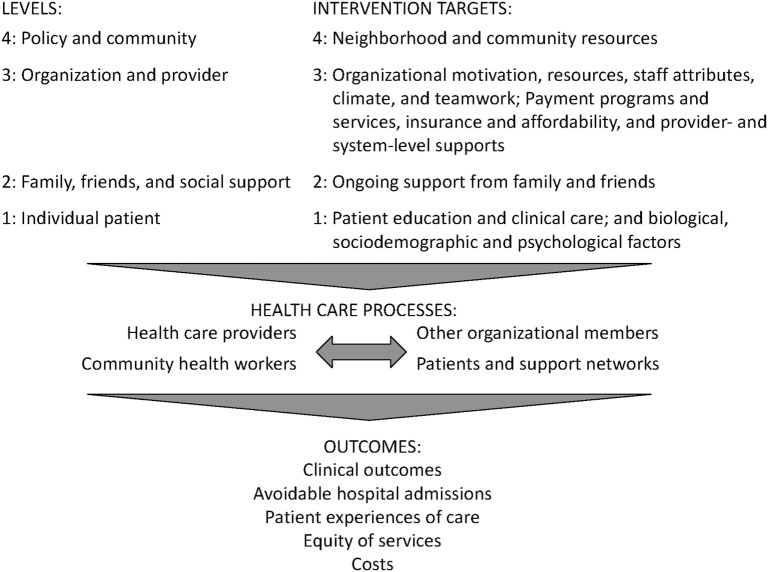
This graphic is derived from the conceptual model, *Factors that influence disparities in access to care and quality of health care services, by level* created from the analysis of findings from systematic reviews published as: Purnell et al. (4).

The reasons for the documented oral health and health care disparities between countries and populations are complicated (2). They are now being understood in terms of ecological models (3, 4) which posit that factors at multiple levels—in the case of our project, community (level 4), site and provider (level 3), family (level 2), and patient (level 1)—influence disparities in access to and quality of services (Figure 2).

As per our multi-level approach, interventions that address factors at multiple levels may be more effective than those that target a single level (5). For instance, remote electronic health record (EHR) data entry at the institutional level will permit tracking and evaluation of our multi-level intervention at the community, site, provider, and patient levels, in accordance with the implementation strategy to change record systems. This will better enable us to determine whether or not our multi-level intervention results in, e.g., improved patient care at the individual level and enhanced oral health at the community level.

In the United States, oral diseases ranging from dental caries to oral cancers cause pain and disability for millions of US children and adults (6). This “silent epidemic” has disproportionately severe impacts on marginalized populations, including people from historically disadvantaged backgrounds and racial/ethnic minorities (7). Further, a thorough oral examination can detect signs of general health problems, including nutritional deficiencies, immune disorders, injuries, and certain cancers, with referrals to apt health care providers where indicated (7). Relatedly, and of critical, but underappreciated importance, there is a segment of the population that visits a dental provider, but not a primary medical provider each year. Using data collected as part of the Medical Expenditure Panel Survey (MEPS), Strauss et al. found that of the 24.1% of adults who do not access general outpatient care, 23.1% visited a dentist (8). Hence, chairside screening for chronic conditions, such as diabetes and hypertension with referrals to primary care providers where indicated has the potential to identify persons who are unaware of their disease status, improve patient health, and lower health care costs (9, 10).

As a result, both oral and general health conditions may be prevented by regular dental visits (6). Accordingly, Healthy People 2020 Leading Health Indicator (meaning that it is a high priority, evidence-based health issue) is Oral Health-7 (OH-7), namely: increase the proportion of children, adolescents, and adults who used the oral health care system in the past years (6). Unfortunately, the US population as a whole has been moving away from the target of 49.0%, from a baseline of 44.5% in 2007 to 42.1% in 2012. For the Asian-only subgroup, the same trend exists, but at a lower prevalence over time, from a baseline of 41.3% in 2007 to 38.2% in 2012 (6).

The oral health benefits of fluoride have been well known for more than 70 years (11). Specifically, fluoride reduces the risk of dental caries in both children and adults through a variety of mechanisms, including: incorporating into enamel before teeth erupt; preventing demineralization and enhancing remineralization of teeth; and inhibiting bacterial activity in dental plaque (7, 12, 13). The Centers for Disease Control and Prevention recommends drinking fluoridated water if it is available and using fluoride toothpaste (12). The American Dental Association (ADA) recommends brushing teeth for 2 min twice a day with a soft-bristled toothbrush and fluoride toothpaste as part of a complete dental care routine (14). A video on proper brushing technique is also available on the ADA website (14).

### Rationale for Prioritizing Asian American Oral Health Research

There are at least four compelling reasons to prioritize Asian American oral health research. First, Asian Americans are the fastest growing minority group in the United States, increasing in size by 43.3% between 2000 and 2010, more than four times faster than the total US population. Nationwide, there are nearly 14.6 million Asian Americans, representing approximately 4.8% of the US population, of whom more than 60% are foreign born and more than 30% have limited English proficiency (15). Second, studies have shown that current research and policy practices give rise to erroneous conclusions about Asian American health due to omission from data collection efforts, aggregation across Asian subgroups, and extrapolation of results from one Asian subgroup to another (16, 17). The preponderance of the limited research is based on West Coast populations, leading to a dearth of understanding of substantial and emerging Asian American communities in the Midwest, Northeast, and Southwest. Third, the “model minority” stereotype of Asian Americans as “wealthier, wiser, and healthier” than other racial/ethnic minority populations undermines the significance of health disparities experienced among and within Asian American communities and the need to devote resources to mitigate those disparities (18). Fourth, findings of community health resources and needs assessments that sampled underserved and hard to reach New York, NY Asian immigrant populations consistently found that oral disease was a top concern for Chinese and Sikh South Indian populations (19–21). Fully 22% of Chinese respondents ranked oral or dental health as their top health concern, 68% rated their oral health as poor or fair, and only 53% reported having received an oral/dental health check-up in the past year (20).

In separate and related research conducted in New York, NY, the self-reported frequency of visiting a dentist in the past year for Chinese respondents ranged dramatically depending upon the sample studied, from 15.7% among participants recruited from recent immigrant enclaves (22) to 89.3% among participants recruited from throughout the five boroughs who were low-income, but highly educated (23). In a prospective study that examined racial/ethnic differences in periodontal disease among participants recruited from six US sites, Chinese participants displayed the highest prevalence of self-reported periodontal disease (39.8%), followed by Blacks (32.0%) and Whites (26.0%), with Hispanics displaying the lowest prevalence (17.4%) (24). Finally, a study conducted in Washington, DC among Chinese, Korean, and Vietnamese Americans found that less acculturated Asian Americans were less likely to receive physical, dental, and eye examinations than those who were more acculturated (25). This may be because there is less emphasis on preventive health care in Asian cultures, or because barriers to health care access may amplify the reluctance for preventive care among Asian Americans (25).

### Adaptation of the Sikh American Families Oral Health Promotion Program

Using a community-based participatory research (CBPR) approach, UNITED SIKHS, a community-based organization that pursues projects for the spiritual, social, and economic empowerment of underprivileged and minority communities (26), New York University School of Medicine (NYU Medicine), City University of New York Prevention Research Center (27), and NYU College of Dentistry (NYU Dentistry) (28) developed, implemented, evaluated, and disseminated the *Sikh American Families Oral Health Promotion Program* (29). Several of the implementation strategies identified by Powell et al. were found to be effective in this earlier effort will be adapted for the proposed project, including conduct a local needs assessment, involve patients and family members, use to train-the-trainer strategies to provide hands-on instruction on proper brushing techniques to community educators, and use a community advisory board (CAB) to provide input and advice on implementation efforts (30). Findings were for Sikh participants with no dental insurance prior to program enrollment (*n* = 58), 81.0% credited the program with helping them obtain insurance for them or their children; for participants with no dentist prior to program enrollment (*n* = 68), 92.6% credited the program with helping them or their children find a local dentist (29).

### Systems Modeling to Understand Dynamic Complexity and Simulate Alternate Scenarios

The term “systems science” is used to refer to the big picture of problem solving, where the problem space is conceptualized as a system of interrelated component parts (31). Both the coherent whole of the system and the relationships among the component parts are critical to the system, as they give rise to emergence, meaning much coming from little (32). Note that emergence occurs when even a relatively simple system generates unexpected amounts of complexity, which cannot be understood without the ability to simulate (32).

In order to improve our mental models of the real world, system scientists have developed and leveraged methods, such as system dynamics (SD), agent-based modeling (ABM), geographic information science, and social network simulation. The practice of systems science modeling is situated amidst an ongoing process of observing the real world, formulating mental models of how it works, setting decision rules to guide behavior, and from these heuristics, making decisions that in turn affect the state of the real world (33).

Interventions often fail or even worsen the problems they are intended to solve due to a lack of understanding of real world structures and dynamic complexity. Among the benefits of systems modeling are iterative practice, participatory potential, and possibility thinking. Best principles and recommendations for advancing implementation science through systems science modeling are summarized below (Table 1), based upon the seminal contributions of thought leaders in the field (34).

**Table 1:** Summary of best principles from systems science for informing the modeling process, recommendations for action by implementation scientists, and key references from contributing thought leaders of systems science [adapted from Ref. (34)].

Best principles	Recommendations	Key references
1. Model the problem, not the system	Conduct formative research; construct models collaboratively in interdisciplinary teams	Sterman (33)
2. Pay attention to what is important, not just what is quantifiable	Use qualitative data to derive causal relationships;be guided by deep thinking and multiple perspectives	Meadows (35)
3. Leverage the utility of models as boundary objects	Create modifiable and accessible representations of models; build trust by representing local knowledge	Black (36)
4. Adopt a portfolio approach to model building	Work in parallel to develop separate, but related models in diverse ways; encourage exploration	Metcalf et al. (37)

As part of a body of research to understand the complex set of causal pathways and time delays that compound health inequities over the life course, our research team developed, refined, and tested a portfolio of systems science models that originated in the *ElderSmile* program in northern Manhattan (38). Despite the time and resources required to ensure a participatory approach to group model building that elicits the knowledge of all team members across disciplines and fields, the simulation models devised may more accurately reflect real world conditions and possibilities. If so, in the end, time and resources will have been well spent in the service of running virtual experiments that may more effectively direct program enhancements and policy changes that improve the health and well-being of disadvantaged adults, and may be adapted for other populations and locales.

### Statement of Compliance

The feasibility and acceptability study will be conducted in accordance with the International Council on Harmonization guidelines for Good Clinical Practice (ICH E6), the Code of Federal Regulations on the Protection of Human Subjects (45 CFR Part 46), and the National Institute of Dental and Craniofacial Research (NIDCR) Clinical Terms of Award. All personnel involved in the conduct of this study have completed human subject’s protection training.

### Potential Risks and Benefits

#### Potential Risks

Despite the enhanced computer security, patient data may be at a higher risk for computer hacking due to remote data entry, leading to a loss of medical record confidentiality.

#### Potential Benefits

Outreach center clients/patients may benefit from the intervention, including the translated and culturally customized literature, by being influenced to pursue dental care and conduct twice daily dental hygiene using evidence-based products and procedures.

### Objectives

The ultimate goal of this study is to provide information for the design and implementation of a randomized controlled trial of a participatory, multi-level, partnered (i.e., with community stakeholders) intervention to improve the oral and general health of low-income Chinese American adults. Toward this end, this study has three objectives.

#### Primary

- To evaluate and enhance the feasibility and acceptability of a partnered intervention designed to improve oral health for low-income, urban Chinese American adults at three community sites.

#### Secondary

- To evaluate and enhance the feasibility and acceptability of using remote entry features of EHR software at NYU Dentistry to enter patient information at three Chinese American community sites.
- To model knowledge held *a priori* by non-patient participants about factors that influence access to oral health care and care-seeking behaviors among low-income, urban Chinese American adults, in order to enhance the intervention during and/or after the study for use in future implementations

## Methods: Frameworks, Setting, Population, and Special Terms

### Theory-Driven Implementation Frameworks

Our study design to implement remote EHR data entry and tracking and a partnering package of evidence-based intervention strategies in diverse Chinese American community outreach sites is guided by two complementary, multi-level frameworks: Consolidated Framework for Implementation Research (CFIR) (39, 40) and Implementation Outcomes Framework (IOF) (41, 42). Specifically, CFIR provides a menu of constructs that have been associated with effective implementation and have been used in a range of applications, including our own oral health research (29, 43–46). For the proposed project, the five domains and the associated constructs that we are particularly interested in exploring are: (1) intervention = partnered EHR enhanced community outreach (adaptability, cost); (2) inner setting = NYU Dentistry *Local Community Outreach Programs* and clinics (implementation climate, relative priority); (3) outer setting = Chinese American outreach sites (patient needs and resources); (4) characteristics of individuals involved = champion (Dr. Wolff), implementation leaders (Drs. Schenkel and Perelman), external change agents (CAB members and site directors), researchers, dental providers, family members, and patients (self-efficacy); and (5) process of implementation (planning, engaging, reflecting, and evaluating). A final critical component of CFIR is the process of adaptation of the intervention for diverse partnering sites.

Implementation Outcomes Framework is clear in distinguishing implementation outcomes (acceptability, adoption, implementation cost, sustainability), service outcomes (effectiveness, equity), and client outcomes (satisfaction), all of which we intend to assess. While IOF provides an evaluation outcomes framework that organizes the multiple facets that affect implementation of new interventions, CFIR provides a framework for understanding the multiple domains that influence implementation and adoption of these interventions.

Finally, because our proposed intervention is both multi-level and dynamic with numerous involved constructs, we intend to model the knowledge gained about factors at the community, site, provider, family, and patient levels to improve oral health using a participatory group modeling approach. We will leverage the power and flexibility of software programs, such as AnyLogic^1^ and Vensim,^2^ to construct simulation models that enable integration of different structural components of models: agents, social networks, geographic information system data, and stock-flow SD.

### Rationale for the Selection of the Setting and Population

The setting for this project is the NYU Dentistry *Local Community Outreach Programs* (20). NYU Dentistry conducts local community outreach in two ways. First, all dental students must complete 4-month long community-based rotations, in which they spend 1-day per week providing direct patient care under faculty supervision in 1 out of 7 locations in four boroughs of New York, NY. A second type of outreach, which is the focus of this research, takes place in dozens of locations throughout New York, NY; it is an entirely voluntary effort shared by faculty preceptors and students. In 2015, the number of volunteer community events reached 126. The volunteer screening events are held an average of three times per week, on weekdays and weekends, with 6–8 students typically taking part in each event. Although they do not directly treat patients at these sites, students refer many of them to NYU Dentistry. To encourage patients to visit a dentist, students provide each patient screened with a voucher worth $205.00 for oral health care at NYU Dentistry to cover his/her comprehensive oral examination, treatment plan, and prophylaxis at no charge and with no co-payment required.

In discussions with Dr. Schenkel, the program leader, we learned that turnout at Chinese sites was especially high, affirming the findings of a recent *Chinese Community Health Resources and Needs Assessment* conducted by NYU Medicine, where oral health was identified as a top concern (20). Dr. Schenkel also expressed a need for data collection and analysis to evaluate the program. With guidance from Dr. Wolff, the project champion, Dr. Perelman, the IT leader, was recruited to plan, implement, and evaluate remote EHR data entry and tracking at community sites.

### Populations/Units of Analysis for the Feasibility and Acceptability Study

As we are utilizing a CBPR approach, several different populations/units of analysis are included in this feasibility and acceptability study:

- In accordance with CBPR principles, we will establish a *CAB* comprised of eight members to guide all aspects of this study. We are currently recruiting CAB members from the following local partners: Chinatown YMCA, Chinatown Planning Council Manhattan, Hamilton-Madison House, and the NYU Medicine *Clinical and Translational Science Institute* Integrating Special Populations Program.
- The *research staff* (*n* = 10) comprises of three Multiple Principal Investigators (MPIs) (Drs. Northridge, Trinh-Shevrin, and Metcalf), the three Co-Investigators (Co-Is) at NYU Medicine (Drs. Troxel, Islam, and Yi), two Project Coordinators (TBN), and two modelers (Ms. Zhang and TBN).
- The *NYU administrators* (*n* = 3) include both Co-Is at NYU Dentistry (Drs. Schenkel and Wolff) and an IT Specialist (Dr. Perelman).
- The *providers* (*n* = 8) include faculty dentists at NYU Dentistry who participate in local community outreach events (*n* = 6) and bilingual (English and Mandarin Chinese) community health workers (CHWs) (*n* = 2).
- *Outreach site directors* at each of three participating community outreach centers (*n* = 3) serving low-income Chinese American populations in New York, NY.
- *EHR patient participants*. At least 50 low-income Chinese American adult patients will undergo a dental screening at each of three community outreach centers (*n* = 150), and have their data remotely entered into the NYU Dentistry EHR.
- *Interview patient participants*. Approximately 30 Chinese American patients screened at each of three community outreach centers will be enrolled in the study as participants (*n* = 90) to complete interviews about their satisfaction with the partnered intervention and their use of dental services and evidence-based oral health behaviors.

### Special Terms

Terms with a special meaning regarding this protocol are explained next.

**Figure 3:**
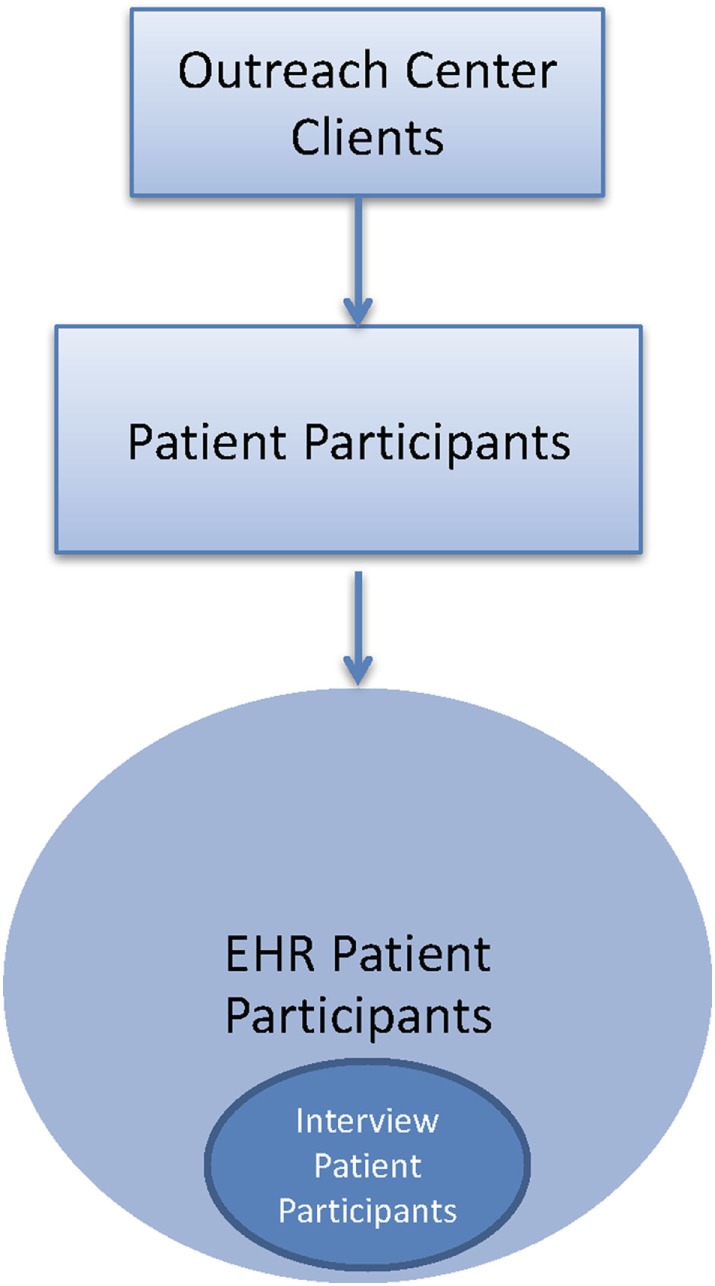
A flow diagram that depicts the patient participants in this study.

**Figure 4:**
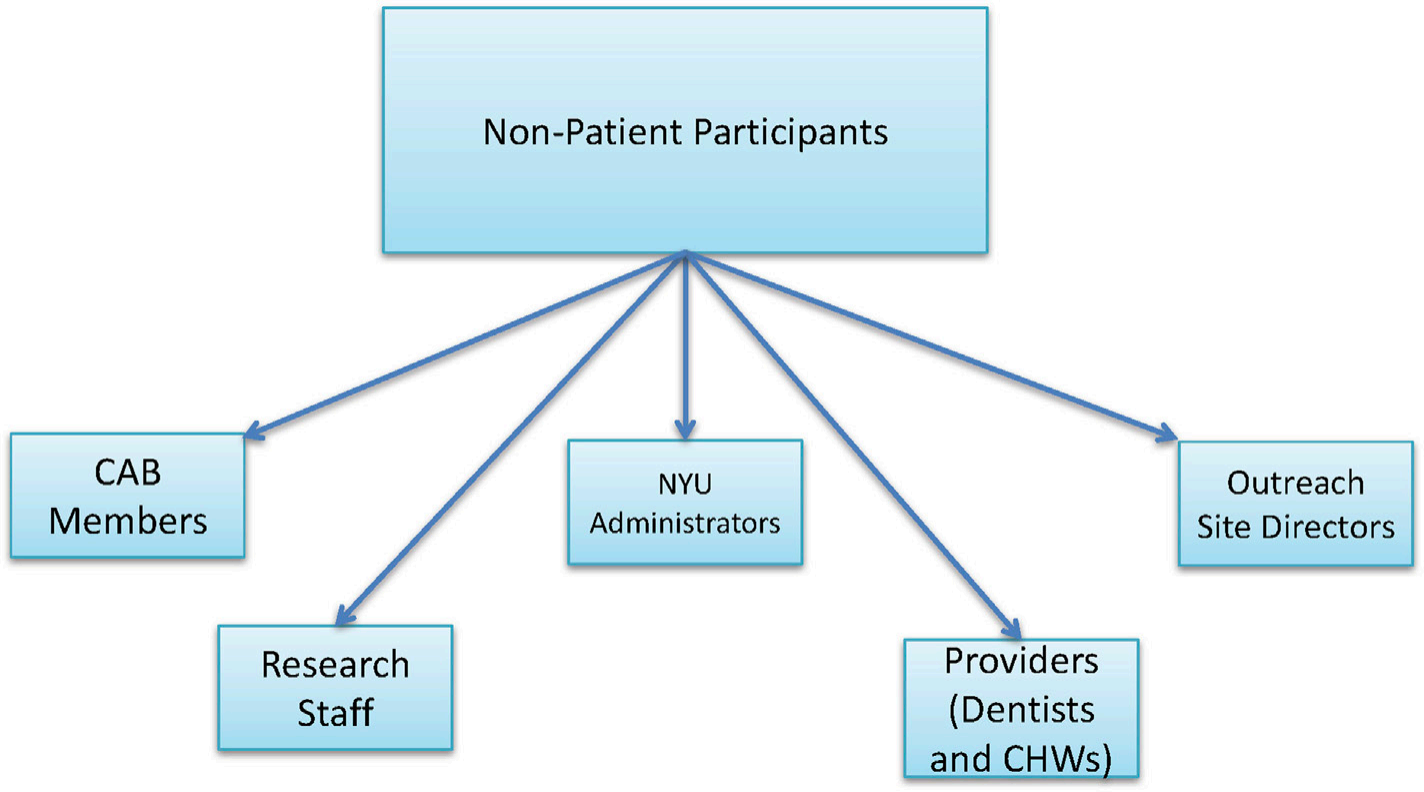
A diagram that depicts the non-patient participants in this study.

- The *partnered intervention* consists of the following four evidence-based strategies: (1) written agreements of collaboration for dental screening at the site level; (2) culturally tailored and language-specific adaptation of materials at the community and site levels; (3) demonstrations with role-playing of proper brushing with fluoride toothpaste and flossing techniques at the site and provider levels; and (4) CHW follow-up with patients about oral health care receipt and dental hygiene behaviors at the family and patient levels.
- *Patient participants* refer to study participants who are also patients screened at the urban outreach centers. Patient participants are first enrolled as EHR patient participants. A subset of the EHR patient participants also complete exit interviews after the intervention at urban outreach centers and are referred to as interview patient participants (Figure 3).
- *Non-patient participants* refer to all other study participants who are not patients screened at the urban outreach centers, including CAB members, research staff, NYU administrators, providers (dentists and CHWs), and outreach site directors (Figure 4).

## Methods: Outcomes, Design, Enrollment, and Withdrawal

### Study Outcome Measures

The primary and secondary outcome measures for this feasibility and acceptability study are provided below.

#### Primary

Patient satisfaction with the partnered intervention components based on exit interviews.

#### Secondary

Table 2 provides the series of secondary outcome measures associated with the objectives of this feasibility and acceptability study.

**Table 2:** List of secondary outcome measures, with their corresponding constructs, levels of analysis, and data sources.

Constructs	Levels of analysis	Measures (Quantitative/Qualitative)	Data sources
**Implementation outcome measures**			
Acceptability	Provider	Satisfaction with the partnering components and perceived ease of use of the remote entry electronic health record (EHR)	Exit interviews with patients; semi-structured interviews
Adoption	Provider institution	Uptake and utilization of remote entry EHR and partnering components by providers and program	Observation; semi-structured interviews; EHR
Costs	Institution	Intervention and implementation costs, including investment, supply, and opportunity costs	Semi-structured interviews; EHR
Feasibility	Provider site	Extent to which the remote EHR entry and partnered intervention model are compatible with resources and training	Semi-structured interviews; EHR
Fidelity	Provider	Adherence to program protocol and quality of delivery	community health worker logs; self-report
Sustainability	Institution site	Sustained remote EHR use at outreach events and partnering package of interventions	Semi-structured interviews; EHR
**Service outcome measures**			
Equity	Community provider family patient	Support from community partners, providers (including NYU Dentistry), family members, and patients to direct resources to less well-served and less well-studied populations (Chinese American adults)	Baseline survey; follow-up patient interviews; semi-structured interviews; EHR
**Organizational characteristics**			
Engagement	Institutionsite	Commitment, involvement, and accountability of leaders with the implementation	Semi-structured interviews

Other important outcomes of this feasibility and acceptability study include:

- Work flow analysis of the interviews of research staff, NYU administrators, and providers (dentists and CHWs) is a secondary measure designed to evaluate and refine the use of the remote EHR.
- Knowledge, expertise, insights, and opinions of the research staff, NYU administrators, providers (dentists and CHWs), outreach site directors, and CAB members about factors that influence access to oral health care and care-seeking behaviors among low-income, urban Chinese American adults are secondary measures designed to be used in simulations to represent dynamics at multiple levels (patient, family, provider, site, and community).
- The simulation modeling platform is a research product of this study that will be used to experiment with strategies to promote preventive and restorative care through regular dental visits and self-efficacy among Chinese Americans.

### Study Design

**Figure 5:**
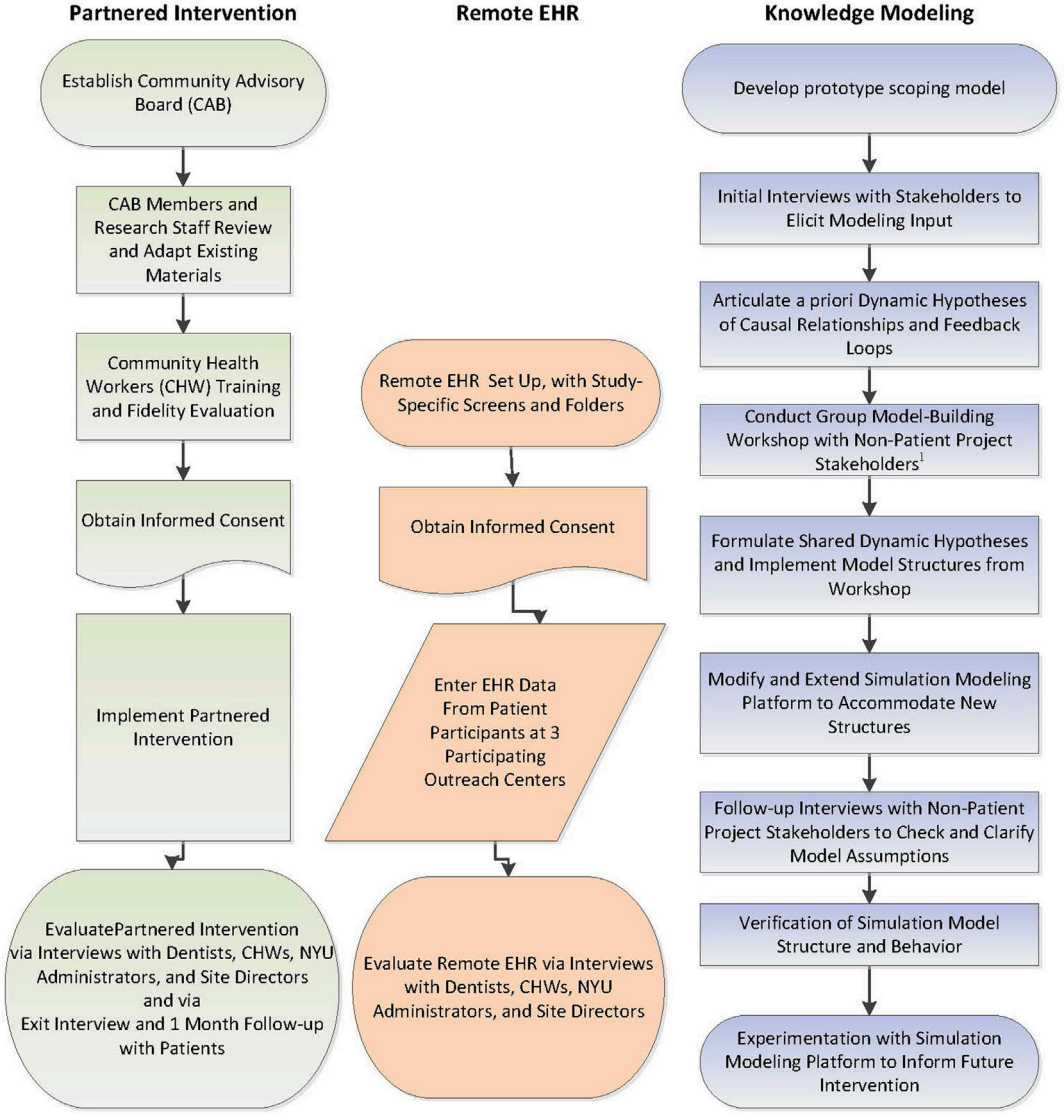
Schematic of the study design involving three related components: partnered intervention, remote electronic health record, and knowledge modeling.

This feasibility and acceptability study will be conducted at three community outreach centers serving an urban, low-income Chinese American population. The study will evaluate the feasibility and acceptability of implementing a partnered intervention to improve the oral and general health of low-income, urban Chinese American adults and of using remote entry into an EHR. The evaluation will include group adaptation sessions and workflow analyses of the EHR implementation, involving multiple recorded sessions with NYU administrators, providers (dentists and CHWs), outreach site directors, and research staff. The study will also model *a priori* knowledge held by non-patient participants to evaluate and enhance the intervention during and/or after the study for use in future implementations (Figure 5).

Approximately 50 patient participants who self-identify as Chinese American from each of three outreach centers (*n* = 150) will be consented to allow the entry of their data (e.g., demographic information, medical history, receipt of oral health care visits, dental hygiene behaviors, and health and health care measures) into the remote EHR by authorized NYU Dentistry staff (EHR patient participants). Of these 150 EHR patient participants, research staff will survey approximately 30 Chinese American patient participants from each of three outreach centers (*n* = 90) regarding their satisfaction with the intervention components (interview patient participants). The study team will also evaluate feedback from approximately 32 non-patient participants selected from the following groups: research staff, CAB members, outreach site directors, NYU administrators, and providers (dentists and CHWs); these individuals will be interviewed about various aspects of the partnered intervention and/or the remote EHR implementation process and/or their *a priori* knowledge of factors that influence access to oral health care and care-seeking behaviors among low-income, urban Chinese American adults.

### Study Enrollment

#### Patient Participants

Outreach center patients will be enrolled into either or both of two groups.

#### Group 1

Approximately 50 patients from each of three centers (*n* = 150) will be consented to allow their data to be entered *via* the remote EHR. These *EHR patient participants* must meet all of the following criteria to be enrolled:

1. Greater than or equal to 21 years of age.
2. Self-identify as being of Chinese ethnicity.
3. Live in any of the five boroughs of New York, NY and visit a participating outreach center.
4. Able and willing to provide informed consent to have their data entered into the remote her.

#### Group 2

Approximately 30 patients from each of three centers (*n* = 90) will be consented to participate in an exit interview and a follow-up interview. These *interview patient participants* must meet all of the following criteria:

1. Greater than or equal to 21 years of age.
2. Self-identify as being of Chinese ethnicity.
3. Live in any of the five boroughs of New York, NY and visit a participating outreach center.
4. Able and willing to provide informed consent and participate in an exit interview and a follow-up interview.

#### Non-Patient Participants

Non-patient participants will be enrolled into either or both of two groups.

#### Group 1

Approximately 20 research staff, NYU administrators, outreach center directors, and providers (dentists and CHWs) will be enrolled to participate in interviews about the partnered intervention and/or remote EHR. These non-patient participants must meet all of the following criteria:

1. Greater than or equal to 18 years of age.
2. Be employed or volunteers at participating outreach centers or employed at NYU.
3. For CHW-staff, speak and read Mandarin Chinese.
4. Able and willing to provide informed consent.

#### Group 2

Approximately 32 non-patient participants research staff, NYU administrators, CAB members, outreach site directors, and providers (dentists and CHWs) will be enrolled to participate in interviews and a group model-building workshop to inform model development by sharing their knowledge about factors that influence access to oral health care and care-seeking behaviors among low-income, urban Chinese American adults. These individuals must meet all of the following criteria:

1. Greater than 18 years of age.
2. Be employed or volunteers at participating outreach centers or employed at NYU.
3. Able and willing to provide informed consent.

#### Subject Exclusion Criteria

Individuals meeting any of the following criteria will not be enrolled as either EHR patient participants or interview patient participants:

1. Have an acute or terminal illness or a serious mental illness or any other severe health condition(s) that might preclude visiting an oral health care provider.
2. Are currently participating in another oral health study.

Individuals meeting any of the following criteria will not be enrolled to complete the interviews about the partnered intervention or remote EHR or to provide input to the knowledge modeling activities:

1. Staff in functional areas that do not directly service patients (e.g., custodial staff).

A patient participant may participate in either the EHR patient participant group only or both patient participant groups (interview patient participants are a subset of EHR patient participants). A non-patient participant may participate in any or all of the non-patient participant data collection activities. Co-participation in activities by any subject is not required.

#### Strategies for Recruitment and Retention

Five Chinese American community-based organizations have already volunteered to participate in this study:

- Asian Americans for Equality.
- Chinatown YMCA, a Branch of the YMCA of Greater New York.
- Chinese American Planning Council.
- Coalition for Asian American Children and Families.
- Hamilton-Madison House.

The three outreach centers for this study will be selected from among the affiliated outreach centers of these organizations. Clients from the outreach centers will be recruited by research staff working with three Chinese American community sites in New York, NY.

Electronic health record patient participants (of whom certain individuals are also interview patient participants) will receive a voucher worth $205 for oral health care at NYU Dentistry to cover his/her comprehensive oral examination, treatment plan, and prophylaxis at no charge and with no co-payment required as compensation for participation.

Non-patient participants—research staff, NYU administrators, providers (dentists and CHWs), outreach site directors, and CAB members—will receive no monetary compensation for their participation in the study, over and above their salaries/stipends.

### Study Withdrawal

#### Reasons for Withdrawal

Any of the various participants (i.e., CAB members, outreach site directors, EHR patient subjects, interview patient subjects, research staff, NYU administrators, dentists, and CHWs) may withdraw from the study at any time. Patients will have the right to refuse to participate without any compromise of their health or dental services. Also, if a participant is uncomfortable during an interview or survey administration, he/she may stop at any time without penalty.

#### Handling of Subject Withdrawals or Subject Discontinuation of Study Intervention

If an EHR patient participant withdraws consent, no further data from that patient will be entered into the EHR for that participant. Depending on the nature of the request to withdraw, it may be necessary to remove existing data for that patient from the EHR.

#### Premature Termination or Suspension of Study

This study has no explicit stopping rules. The study may be suspended or prematurely terminated if there is sufficient reasonable cause. Written notification, documenting the reason for study suspension or termination, will be provided by the suspending or terminating party to the MPIs and/or the NIDCR, as applicable. If the study is prematurely terminated or suspended, the MPIs will promptly inform the Institutional Review Boards (IRBs) and provide the reason(s) for the termination or suspension.

Circumstances that may warrant termination include, but are not limited to:

- Determination of an unexpected, significant, or unacceptable risk to participants.
- Insufficient adherence to protocol requirements.
- Data that are not sufficiently complete and/or evaluable.

## Methods: Intervention, Training, Schedule, and Activities

### Administration of Intervention

Initially, a CAB will be established to guide all aspects of the study.

#### Partnered Intervention

Our partnering package of interventions builds upon the evidence-based practices of the NYU Dentistry *Local Community Outreach Programs* and the results of our pilot study in the Sikh American community (20), and aligns with the implementation strategies from the *Expert Recommendations for Implementing Change* (EPIC) project (30). We will work closely with the directors of three Chinese American sites to create written agreements of collaboration that outline the roles and responsibilities of the investigative team and the sites. An integral part of effectively implementing oral health activities with low income, racial and ethnic minority, and immigrant populations is to develop program materials that are specific to the local community (47). Training lay individuals of the same cultural and linguistic background as participants, e.g., CHWs through train-the-trainer techniques, has been found to be an acceptable approach for delivering culturally appropriate, community-based oral health interventions, as well as for recruiting participants into interventions through community and social networks (48–51). CHWs have been found to be effective in providing dental education and counseling (52), leading interactive demonstrations of brushing with fluoride toothpaste and flossing (53, 54), and improving access to dental care through dental coverage and linkage to local dentists (20).

#### Development of Culturally Tailored and Language-Specific Materials

The CAB will be responsible for reviewing existing program materials as an integral part of adapting them for the local Chinese American population. This will entail a multi-step process. Existing English and simplified Mandarin Chinese language materials will be presented to the CAB. Dr. Yi will then lead a guided discussion structured around the 4 P’s of social marketing. For product, CAB members will be asked if the materials encourage prevention of oral conditions through regular dental visits and brushing with fluoride toothpaste. For price, CAB members will be asked how much it will cost a person to take on the desired behaviors in terms of time and effort, not merely dollars and cents. For place, CAB members will be asked to help to compile a list of local dental providers in addition to NYU Dentistry who provide culturally tailored and language-specific oral health care to Chinese American families. For promotion, CAB members will be asked to identify other Chinese American community change agents to promote the program through word of mouth, social media, and neighborhood venues. CAB input will also be sought on incorporating appropriate imagery and cultural beliefs regarding oral health in the Chinese American community. This guidance will then be used to adapt both print and online materials. Finally, the adapted materials will be presented back to the CAB to ensure their input was accurately captured.

### Procedures for Training Interventionists and Monitoring Intervention Fidelity

Both CHWs in this study were formerly trained as CHWs, have a history of engaging in health promotion with the Chinese American community, and are bilingual in English and Mandarin Chinese (the primary dialect of participating community outreach sites). Specifically, the project CHWs were previously trained in a core competency program that employed diverse training methods, guided by adult learning principles and popular education philosophy. We will further train the project CHWs in the oral health promotion demonstration protocol and on oral health services and programs available at local clinics and hospitals. The investigative team and CAB members will also collect, assess, and deliver to the CHWs updated information regarding health and dental insurance and access to oral health programs available for low-income and immigrant communities. NYU Dentistry investigators and staff will train the CHWs using models on evidence-based oral health practices, stressing the importance of drinking fluoridated water and brushing teeth for 2 min twice a day with a soft-bristled toothbrush and fluoride toothpaste (“painting the teeth with fluoride”). This train-the-trainer model will promote peer support and allow the project to be replicated and sustained across settings. Interactive educational techniques will be integrated into the demonstrations. As in our pilot work (20), hands-on instruction will be provided on proper brushing and flossing techniques, culturally tailored health promotion methods (i.e., preparing healthy traditional meals and using the plate method to determine the proper balance and size of portions), and goal-setting skills. Trainees will then demonstrate the presented procedures back to the trainers using models.

At the end of the training, the CHWs will collaborate in groups to practice delivering short excerpts from the curriculum to their peers and project team members, with the trainers providing comments and assistance. Approximately, 1-month before the CHW training is complete, the curriculum will be pilot tested to ensure its cultural appropriateness with patients. Two mock educational sessions and a final examination of knowledge and evaluation of trial encounters with mock participants will be conducted with project investigators and CAB members. Individuals who score below the threshold level of knowledge regarding oral health promotion will receive intensive 1-on-1 tutoring and be required to take a second examination of knowledge. Quality assurance controls will be built into the intervention. Drs. Northridge and Yi will meet with the CHWs on an approximately bi-weekly basis to ensure that the model components are being consistently applied. Each CHW will keep a log of activities and communication around their follow-up of patients. These logs will be reviewed as necessary to evaluate the type and nature of communications between the CHWs and their assigned study participants.

### Study Schedule

The study will extend for approximately 1 year. An approximate timeline for implementation of the various aspects of this study is provided below in Table 3.

**Table 3:** Timeline of study activities.

Study activities	Months 1–6	Months 7–12
Recruitment of Community Advisory Board members and outreach sites		
Review, update, and finalize community health worker (CHW) training materials/Gain Institutional Review Board (IRB) approvals		
Train CHWs, evaluate fidelity, retrain as necessary		
Implement partnered intervention		
*Exit and 1-month follow-up interviews of enrolled interview patients who participated in the partnered intervention*		
*Semi-structured interviews of non-patient participants regarding partnered intervention (including materials review prior to implementation)*		
Workflow analysis for remote electronic health record (EHR) entry		
Pilot testing and live-usability for remote EHR		
*Semi-structured interviews of non-patient participants in EHR implementation*		
*Semi-structured interviews of non-patient participants to inform model*		
*Group model-building workshop*		
Development of simulation modeling platform		
*Data collection activity*		

*The gray shades denote the time period in which the activities will take place*.

### Study Activities by Phase

#### Pre-Intervention Activities

- Create written agreements of collaboration that outline the roles and responsibilities of the investigative team and the outreach sites.
- Develop culturally tailored and language-specific program materials.
- Train CHWs in the intervention protocol.
- Conduct a workflow analysis of remote EHR data entry at Chinese American outreach centers.

#### Outreach Center Activities

- Health Insurance Portability and Accountability Act (HIPAA) certified research staff members or volunteers will explain the consent form, confidentiality agreement, and liability release to each potential patient participant and obtain his/her signature.
- Remote EHR Data Collection and Dental Screening:
- Authorized NYU Dentistry personnel will directly enter the demographic information of patient participants into the customized EHR using the study-specific SCREENING option.
- EHR patient participants will complete a short paper intake form that will be scanned directly into a designated SCREENING folder in the EHR regarding medical conditions that could affect, or be affected by, their oral health (Yes/No checklist) and a brief questionnaire on self-reported receipt of a dental visit in the past year and dental hygiene behaviors (*Oral Health Survey*). These questions will be based on the World Health Organization Oral Health Questionnaire for Adults: (1) How often do you clean your teeth? (Never, Once a month, 2–3 times a month, Once a week, 2–6 times a week, Once a day, Twice or more a day); (2) Do you use any of the following to clean your teeth? Toothbrush, Wooden toothpicks, Plastic toothpicks, Thread (dental floss), Charcoal, Chewstick/miswak, and Others (Please specify); (3) For those who nominate Toothbrush: what type of toothbrush do you use? (Hard-bristled, Medium-bristled, Soft-bristled) (4) Do you use toothpaste to clean your teeth? (5) For those who nominate Toothpaste, Do you use a toothpaste that contains fluoride? Correct responses to all five questions will indicate that EHR patient participants are following the guidance of the ADA on brushing with fluoride toothpaste. These measures will be collected in person *via* self-report to ascertain baseline status at the beginning of each screening event and scanned directly into the NYU Dentistry remote EHR customized for community outreach events.
- An NYU Dentistry faculty dentist will conduct a head and neck/oral examination on each EHR patient participant and directly enter the results into the customized HER.
- The dentist will review the examination results with the EHR patient participant using a customized walkout statement with screening results and follow-up notes and answer any questions regarding oral health concerns.

*Community health workers initial intervention*: trained CHWs will deliver a culturally tailored and language-specific oral health promotion program focusing on demonstrations with role playing of proper brushing with fluoride toothpaste and flossing techniques. They will also provide culturally customized literature to the patients.

*Acceptability data collection*: research staff will conduct a brief exit interview with each interview patient participant regarding acceptability of the intervention and self-efficacy around oral health behaviors (29) using previously validated instruments, requesting permission to contact his/her regular dental provider regarding receipt of a follow-up dental visit and providing a voucher worth $205.00 for oral health care at NYU Dentistry to cover her/his comprehensive oral examination, treatment plan, and prophylaxis at no charge and with no co-payment required. The questions for this survey will be based on our previous oral health promotion program in the Sikh American community to assess acceptability (20). Prior to finalizing the survey for distribution to the interview patient participants, CAB members and research staff will adapt the questions as deemed appropriate for the Chinese American community and to be consistent with the ADA guidance on brushing with fluoride toothpaste.

*Feasibility data collection*: we will also develop a checklist of 10 key components based on process (e.g., patient engagement) and the curriculum (e.g., topics covered) and ask if each one was covered. Endorsement of 8 of the 10 checklist items (80%) by the non-patient participants will be considered as the bar for success for feasibility. Finally, we will allow for open-ended collection of feedback on the feasibility of each of the partnered program components.

#### CHW Follow-up Contact with Interview Patient Participants (Feasibility Data Collection)

Community health worker follow-up of oral health care receipt and dental hygiene behaviors will occur at approximately 1 month (window of −7 days to +1 month) after the partnered intervention. This contact may be *via* telephone or in person.

*Feasibility data collection*: during this contact, the CHWs will assess whether or not each interview patient participant has received or has scheduled a dental visit. The CHWs will also inquire about use of fluoride toothpaste and frequency of teeth brushing, since the last visit in a modified Oral Health Survey.

If no visit has occurred or been scheduled, the CHW will offer to help schedule a dental visit for the interview patient participant or her/his family members at NYU Dentistry, her/his regular dentist, or one of the project-approved local oral health care providers.

#### Post-Intervention Activities

- *Feasibility and acceptability data collection*: semi-structured interviews with participating dentists, CHWs, and other non-patient participants will be conducted.
- *Feasibility data collection*: EHR data will be reviewed to assess feasibility.

#### Knowledge Modeling Activities

- Create the interview guide for non-patient participant input into the modeling (pre-intervention).
- Conduct initial non-patient participant interviews to inform model development.
- Conduct the group model-building workshop.
- Conduct follow-up non-patient participant interviews to clarify assumptions.

## Methods: Procedures, Evaluations, and Statistical Considerations

### Workflow Analysis

The team will conduct a workflow analysis, adapted from the Agency for Healthcare Research and Quality recommendations on workflow assessments (55), which includes:

1. Direct entry of patient demographic and appointment (site) information into the EHR by patient service representatives or other authorized users;
2. Ability to scan patient medical history (plus baseline oral health measures of self-reported receipt of a dental visit in the past year and brushing with fluoride toothpaste as recommended) directly into a designated SCREENING folder, followed by transcription and review by faculty dentists;
3. MiFi (Wi-Fi hotspot) configuration to each specific laptop that will only allow EHR access to the matching laptop;
4. Multiple layers of security embedded to comply with all applicable policies;
5. Dual authentication process through Citrix *via* NYU active directory followed by login to the EHR *via* NYU ID card;
6. Direct entry of screening results into the EHR by dentists; and
7. Customized walkout statements for interview patient participants with screening results and follow-up notes.

A matrix will be created with three categories: people, documents, and information content. The group adaptation session will be guided by a user-centered design facilitation protocol that sequentially leads the group through presentation of specific remote EHR use cases that include variations on the original EHR data entry screens adapted to the workflow characteristics of local community sites. For each presented use case, the group discussion will focus on the workflow at the site around the role responsibilities (people), documents, and information content (patient medical history, self-reported outcome measures, head and neck/oral examination results) with regards to reviewing customized EHR protocols based on the findings. The discussion will be digitally recorded and each non-patient participant will be given color-coded response sheets to record their perspectives on how to enhance the usefulness of the customized EHR protocols within the community outreach site setting. The digital recordings and response sheets will then be processed, summarized, and converted into adaptation recommendations by the study team.

Next, the study team will transform the recommendations into proposed revisions and document them in revised standard workflow diagrams that build on established workflows to minimize changes at the sites or new work for the dental providers. The insights will then be used to adapt the customized EHR protocols and related workflows, and will be validated in a follow-up group meeting at each site where the adapted EHR screens will be presented and assessed according to the IOF implementation outcome measures of acceptability and adoption. During these follow-up meetings, we will identify any additional workflow variations that the EHR protocols may need to support. Candidate workflows will then be discussed with the project team and other non-patient participants to finalize the adapted workflow integration approach.

### Pilot Testing and Live Usability for Remote EHR

In order to account for real world conditions, the intervention will be pilot tested at three Chinese American sites before rolling it out to additional study sites during a planned randomized controlled trial. Early formative observations/short interviews will be conducted with dental teams at each of the pilot sites regarding interaction with the customized EHR. This pilot testing will examine impact on workflow, uncover any new usability problems, and identify any educational needs to be included before large-scale implementation. As providers at the pilot sites engage with the EHR, live-usability testing will be conducted, consisting of direct observations by the research team. Live usability is ideal for observing the impact of new tools on real setting workflows and for observing alternative workflows that can be missed during simulations.

### Semi-Structured Interviews Regarding the Remote EHR and Partnered Intervention

Semi-structured interviews will be conducted with participating dentists, CHWs, and other non-patient participants in administrative and technical roles after the intervention. We anticipate conducting approximately 20 interviews before obtaining data saturation. The interviews will be informed by the CFIR and IOF constructs and will assess specific barriers to sustaining the partnered intervention and strategies for addressing those barriers to facilitate integration of the intervention into the routine workflow of the NYU Dentistry Local Community Outreach Programs.

In addition to survey questions, acceptability will also be assessed using open-ended questions, such as:

- Please tell us what you liked most about the program.
- Please tell us what you did not like about the program.
- Please tell us what changes can be made to improve the program.
- Overall program satisfaction (scale of 1–10 answer range).
- How did you feel about the topics covered?
- How did you feel about the length of time of the dental screening and oral health promotion demonstrations?

Feasibility will be assessed among the dentists, CHWs, NYU administrators, and site directors at the three partnering organizations. The following questions will be asked:

- How did you feel about the intake procedures?
- How did you feel about the oral health demonstrations by CHWs?
- How did you feel about the length of time of each dental screening?

### Participatory Modeling of Non-Patient Participant Knowledge

The third aim of this study is to model knowledge held by non-patient participants about factors that influence access to oral health care and care-seeking behaviors among low-income, urban Chinese American adults. This information will be used in designing simulation models at multiple levels, from multiple perspectives. A systems science approach will be undertaken to integrate knowledge held by non-patient participants into simulation models to explore alternative paths toward improved health and health care for low-income, urban Chinese American adults *via* community-based outreach followed by clinical care. These simulation models will be designed using a multi-method approach, in which principles of SD are used to incorporate feedback effects and delays through stocks that accumulate flows (rates of change over time). The SD approach will be integrated with an ABM framework that is used to appropriately represent dynamics at the community, site, provider, family, and patient levels. The model platform developed for this study will contain multiple model structures that characterize different dynamics and reflect participant input.

#### Modeling to Anticipate Effects of Interventions

The models in this platform will simulate implications of hypotheses elicited *a priori* (before implementation of the partnered intervention) from non-patient participants. The *a priori* model platform will enable comparison to models that are later developed with hindsight from implementation of the remote EHR and partnered interventions. This model platform will therefore test the relative effectiveness of the interventions as anticipated under these *a priori* assumptions. Toward this end, this effort will involve design of scoping models that establish a baseline for simulating access at the community level and care-seeking behaviors at the individual level.

A participatory SD modeling process will be undertaken *via* a group model-building workshop held with non-patient participants as well as semi-structured interviews with individual non-patient participants to elicit targeted model input and feedback on assumptions. The UB Geography Systems Science Modeling Team will work closely with non-patient participants to devise indices for input parameters and indicators for outcomes of simulation experiments. In addition to informing the design of model structures, this participatory approach will enable non-patient participants to better assess the results of the simulation models developed in this *a priori* model platform for authenticity and identification of insights for subsequent implementation research. The resulting model platform will establish a multi-level agent-based GIS framework for simulation modeling of access to oral health care and care-seeking behaviors by low-income, urban Chinese American adults at the community, site, provider, family, and patient levels.

### Study Hypotheses

Our hypotheses for this feasibility and acceptability study are stated next.

#### Primary

Based on exit interviews, patient participants in this study will be satisfied with the partnered intervention components.

#### Secondary

- Based on semi-structured interviews, non-patient participants in the study will be satisfied with the partnered intervention components and remote entry features of EHR software at NYU Dentistry to enter patient information at three Chinese American community sites.
- The knowledge held *a priori* by non-patient participants about factors that influence access to oral health care and care-seeking behaviors among low-income, urban Chinese American adults will enhance the intervention during and/or after the study for further use in future implementations.

### Sample Size Considerations

No formal sample size estimates were performed for this feasibility and acceptability study. The bar for success for both feasibility and acceptability is 80% of enrolled patient and non-patient participants report being satisfied or very satisfied with the partnered intervention components.

### Planned Interim Analyses

Because this is a feasibility and acceptability study, there will be interim reviews of interview data in order to modify aspects of the partnered intervention and the remote EHR processes during the course of the study.

### Final Analysis Plan

Acceptability of the partnered intervention will be assessed through exit interviews of the interview patient participants.

As we did with the *Sikh American Families Oral Health Promotion Program* (29), we will utilize a pre-post retrospective evaluation design. In this format, all questions will be asked in a single exit interview, but where applicable, will use the phrasing, “Prior to the beginning of the program…” followed by, “At the present time…” Table 4 provides the measures and definitions of the oral health promotion, self-efficacy, and acceptability measures used in our prior research with the Sikh American community that will be adapted for the present feasibility and acceptability study with the Chinese American community.

**Table 4:** Measures and definitions of oral health promotion, self-efficacy, and acceptability measures.

Question/domain	Observed change in the Sikh American Program	Expected absolute percent change	Expected relative percent change^a^
How often do you brush your teeth for at least two minutes?	Increase from 17.9 to 64.7% of those reporting “More than once a day”	+45%	+261%

How often do you floss?	Increase from 4.4 to 22.1% of those reporting “More than once a day”	+20%	+400%

How confident… (a) Do you know how to take good care of your mouth, teeth, and gums? (b) Do you feel asking your dentist or oral hygienist questions?	(a) Increase from 0% to 65.7% of those reporting “Very confident” (b) Increase from 7.4% to 75% of those reporting “Very confident”	(a) +65% (b) +65%	(a) +6,500% (b) +864%

Agreement with the following statements: (a) Community health worker (CHW) answered my concerns and questions (b) CHW helped me to improve how I take care of my health (c) Information and topics were informative (d) In-person demonstrations helpful in improving oral health	Reported “Strongly agree” (a) 57.4% (b) 60.3% (c) 69.1% (d) 76.1%	N/A	N/A

*^a^We additionally list relative percent change in the case that baseline behaviors among the Chinese American community are very different from those in the Sikh American community*.

The percent change from pre-post will be compared using *t*-tests for proportions. Given that many of the answer choices are non-binary, we will also compare the shift of responses from pre-post across the categories of response using chi-squared tests. The threshold of success for acceptability of the partnered intervention will be that 80% or more of interview patient participants rate all four acceptability questions as agree or strongly agree.

Note that this is not an exhaustive list of questions that will be asked; we are simply highlighting those questions that we will use to quantify acceptability. The full planned questionnaire is attached as an Appendix.

#### Health Care Utilization and Oral Health Promotion Measures

Our ultimate health care utilization measure of interest is receipt of a dental visit within the last 12 months. This will be measured using the MEPS definition, where dental visit refers to care by or visits to any type of dental provider. This will allow for direct comparison with Healthy People 2020 Leading Health Indicator OH-7 to increase the proportion of children, adolescents, and adults who used the oral health care system in the past year.

Our central oral health promotion measure is self-reported brushing of teeth for 2 min twice a day with a soft-bristled toothbrush and fluoride toothpaste at the interview patient participant’s 1-month follow-up visit. For the primary health care utilization measure of receipt of a dental visit within the last 12 months, we will also access the NYU Dentistry EHR database and follow-up with oral health care providers identified by participants in HIPAA approved procedures to ascertain receipt of a dental visit in the last 12 months.

### Source Documents and Access to Source Data/Documents

Study staff will maintain appropriate research records for this study, in compliance with ICH E6, Section 4.9 and regulatory and institutional requirements for the protection of confidentiality of subjects. Study staff will permit authorized representatives of NIDCR to examine (and when required by applicable law, to copy) research records for the purposes of quality assurance reviews, audits, and evaluation of the study safety, progress, and data validity. Patient participant data will be remotely entered directly into the EHR.

### Quality Control and Quality Assurance

The MPIs will be responsible for ensuring that the study is conducted according to the protocol and ensuring data integrity. The MPIs will review the data for safety concerns and data trends at regular intervals, and will promptly report to the IRB and NIDCR any Unanticipated Problem (UP), protocol deviation, or any other significant event that arises during the conduct of the study.

## Assessment of Safety

### Specification of Safety Parameters

Safety monitoring for this study will focus on UPs involving risks to subjects, including UPs that meet the definition of a serious adverse event.

#### Unanticipated Problems

The Office for Human Research Protections (OHRP) considers UPs involving risks to subjects or others to include, in general, any incident, experience, or outcome that meets all of the following criteria: unexpected in terms of nature, severity, or frequency given, (a) the research procedures that are described in the protocol-related documents, such as the IRB-approved research protocol and informed consent document; and (b) the characteristics of the subject population being studied; related or possibly related to participation in the research (“possibly related” means there is a reasonable possibility that the incident, experience, or outcome may have been caused by the procedures involved in the research); and suggests that the research places, subjects, or others at a greater risk of harm (including physical, psychological, economic, or social harm) than was previously known or recognized.

#### UPs Reporting to IRB and NIDCR

Incidents or events that meet the OHRP criteria for UPs require the creation and completion of an UP report form. OHRP recommends that investigators include the following information when reporting an adverse event, or any other incident, experience, or outcome as an UP to the IRB:

- appropriate identifying information for the research protocol, such as the title, investigator’s name, and the IRB project number;
- a detailed description of the adverse event, incident, experience, or outcome;
- an explanation of the basis for determining that the adverse event, incident, experience, or outcome represents an UP;
- a description of any changes to the protocol or other corrective actions that have been taken or are proposed in response to the UP.

To satisfy the requirement for prompt reporting, UPs will be reported using the following timeline:

- UPs that are serious adverse events will be reported to the IRB and to NIDCR within 1 week of the investigator becoming aware of the event.
- Any other UP will be reported to the IRB and to NIDCR within 2 weeks of the investigator becoming aware of the problem.

All UPs should be reported to appropriate institutional officials (as required by an institution’s written reporting procedures), the supporting agency head (or designee), and OHRP within 1 month of the IRB’s receipt of the report of the problem from the investigator.

All UPs will be reported to NIDCR’s centralized reporting system *via* Rho Product Safety:

Product Safety Fax Line (US): (888) 746-3293.
Product Safety Fax Line (International): (919) 287-3998.
Product Safety Email: rho_productsafety@rhoworld.com.
General questions about SAE reporting can be directed to the Rho Product Safety Help Line (available 8:00 a.m.–5:00 p.m. Eastern Time):
US: (888) 746-7231.
International: (919) 595-6486.

### Halting Rules

This study includes no halting rules.

### Study Oversight

The MPIs are responsible for study oversight, in collaboration with the NIDCR Program Official.

## Ethics/Dissemination

### Ethical Standard

The MPIs will ensure that this study is conducted in full conformity with the principles set forth in *The Belmont Report: Ethical Principles and Guidelines for the Protection of Human Subjects of Research*, as drafted by the US National Commission for the Protection of Human Subjects of Biomedical and Behavioral Research (April 18, 1979) and codified in 45 CFR Part 46 and/or the ICH E6.

### Institutional Review Boards

The protocol, informed consent form(s), and all patient participant and non-patient participant materials were submitted to the IRBs at both NYU Langone Health (study i17-01077) and The State University of New York at Buffalo (study 1749) for review and approval. Approval of both the protocol and the consent forms will be obtained before any patient participant or non-patient participant is enrolled. Any amendment to the protocol will require review and approval by the associated IRBs before the changes are implemented in the study.

### Informed Consent Process

Informed consent is a process that is initiated prior to the individual agreeing to participate in the study and continues throughout study participation. Extensive discussion of risks and possible benefits of study participation will be provided to patient participants and their families, if applicable. A consent form describing in detail the study procedures and risks will be given to the patient participant in English or Mandarin Chinese (primary dialect of participating community sites). Consent forms will be IRB-approved, and the patient participant is required to read and review the document or have the document read to him or her. The investigator or designee will explain the research study to the patient participant and answer any questions that may arise. The patient participant will sign the informed consent document prior to any study-related assessments or procedures. Patient participants will be given the opportunity to discuss the study with their surrogates or think about it prior to agreeing to participate. They may withdraw consent at any time throughout the course of the study. A copy of the signed informed consent document will be given to patient participants for their records. The rights and welfare of the patient participants will be protected by emphasizing to them that the quality of their clinical care will not be adversely affected if they decline to participate in this study. The consent process will be documented in the clinical or research record.

Consent for non-patient participant interviews for the knowledge modeling will take place where the interview happens, verbally over the telephone. Dr. Metcalf will send the consent information sheet by email when scheduling the interview, which will be at least 2 days in advance of the interview. To ensuring ongoing consent for follow-up interviews, Dr. Metcalf will remind the non-patient participants about their previous consent and will resend the consent document if needed. Dr. Metcalf will obtain verbal consent for any subsequent recording of information collected during follow-up interviews. Dr. Metcalf will send the consent document ahead of time. Before recording, Dr. Metcalf will ask: have you reviewed the information? Do you have any questions? If the non-patient participant answers “yes” to the first question and “no” to the second question, then she will ask: is it OK if we start the interview now? And if recording the telephone call: is it OK if I begin audio recording? After the participant answers “yes,” the interview will begin.

### Women, Minorities, and Children

The proposed study will enroll Chinese American adults aged 21 years and older. No children will be included since there are separate and targeted NYU Dentistry programs for this age group. The study population is Chinese American adults living in any of the five boroughs of New York, NY. We estimate that approximately 60% of our enrolled patient participants will be women, based on our experience with conducting community-based screening events. All enrolled patient participants will be of self-reported Chinese ethnicity, to ensure that our partnerships and materials are culturally and linguistically relevant. Study sites will be concentrated in lower Manhattan (Chinatown and the Lower East Side) and the Sunset Park area of Brooklyn, which include dense ethnic enclaves of Chinese Americans.

### Subject Confidentiality

Subject confidentiality is strictly held in trust by the MPIs, Co-Is, study staff, and the sponsor(s) and their agents. This confidentiality is extended to cover testing of biological samples and genetic tests in addition to any study information relating to subjects.

The study protocol, documentation, data, and all other information generated will be held in strict confidence. No information concerning the study or the data will be released to any unauthorized third party without prior written approval of the sponsor.

The study monitor or other authorized representatives of the sponsor may inspect all study documents and records required to be maintained by the MPIs, including but not limited to medical records (office, clinic, or hospital) for the study subjects. The modeling site will permit access to such records.

### Certificate of Confidentiality

To further protect the privacy of study subjects, a Certificate of Confidentiality will be obtained from the US National Institutes of Health (NIH). This certificate protects identifiable research information from forced disclosure. It allows the MPIs and others who have access to research records to refuse to disclose identifying information on research participation in any civil, criminal, administrative, legislative, or other proceeding, whether at the federal, state, or local level. By protecting researchers and institutions from being compelled to disclose information that would identify research subjects, Certificates of Confidentiality help achieve the research objectives and promote participation in studies by helping assure confidentiality and privacy to subjects.

### Data Handling and Record Keeping

The MPIs are responsible for ensuring the accuracy, completeness, legibility, and timeliness of the data reported. All source documents will be completed in a neat, legible manner to ensure accurate interpretation of data. The MPIs will maintain adequate case histories of study patient participants and non-patient participants, including accurate case report forms, and source documentation.

The remote EHR entry of data will be protected by means of a dual authentication process through Citrix *via* NYU active directory followed by login to the EHR *via* NYU ID card.

### Data Management Responsibilities

Data collection and accurate documentation are the responsibility of the study staff under the supervision of the MPIs. All source documents and laboratory reports must be reviewed by the study team and data entry staff, which will ensure that they are accurate and complete. UPs and adverse events must be reviewed by the MPIs or their designees.

### Data Capture Methods

Patient participant data will be entered remotely into the EHR. Other data will be collected on paper forms and/or digitally recorded.

### Types of Data

Patient participant data will be captured in the EHR. Data from interviews of patients, research staff, NYU administrators, and providers (dentists and CHWs) will also be captured.

### Study Records Retention

Study records will be maintained for at least 3 years from the date that the grant federal financial report is submitted to the NIH. Study documents will be retained for a minimum of 2 years after the last approval of a marketing application in an ICH region and until there are no pending or contemplated marketing applications in an ICH region or until at least 2 years have elapsed since the formal discontinuation of clinical development of the investigational product. These documents will be retained for a longer period, however, if required by local regulations. No records will be destroyed without the written consent of the sponsor, if applicable. It is the responsibility of the sponsor to inform the MPIs when these documents no longer need to be retained.

### Protocol Deviations

A protocol deviation is any noncompliance with the clinical study protocol, Good Clinical Practice, or Manual of Procedures requirements. The noncompliance may be on the part of the subject, the investigator, or study staff. As a result of deviations, corrective actions are to be developed by the study staff and implemented promptly.

These practices are consistent with investigator and sponsor obligations in ICH E6:

- Compliance with Protocol, Sections 4.5.1, 4.5.2, 4.5.3, and 4.5.4.
- Quality Assurance and Quality Control, Section 5.1.1.
- Noncompliance, Sections 5.20.1 and 5.20.2.

All deviations from the protocol must be addressed in study subject source documents and promptly reported to NIDCR and the local IRB, according to their requirements.

### Publication/Data Sharing Policy

This study will comply with the *NIH Public Access Policy*, which ensures that the public has access to the published results of NIH funded research. It requires scientists to submit final peer-reviewed journal manuscripts that arise from NIH funds to the digital archive PubMed Central upon acceptance for publication.

The International Committee of Medical Journal Editors (ICMJE) member journals have adopted a clinical trials registration policy as a condition for publication. The ICMJE defines a clinical trial as any research project that prospectively assigns human subjects to intervention or concurrent comparison or control groups to study the cause-and-effect relationship between a medical intervention and a health outcome. Medical interventions include drugs, surgical procedures, devices, behavioral treatments, process-of-care changes, and the like. Health outcomes include any biomedical or health-related measures obtained in patients or participants, including pharmacokinetic measures and adverse events. The ICMJE policy requires that all clinical trials be registered in a public trials registry, such as *ClinicalTrials.gov*, which is sponsored by the National Library of Medicine. Other biomedical journals are considering adopting similar policies. For interventional clinical trials performed under NIDCR grants and cooperative agreements, it is the grantee’s responsibility to register the trial in an acceptable registry, so the research results may be considered for publication in ICMJE member journals. The ICMJE does not review specific studies to determine whether registration is necessary; instead, the committee recommends that researchers who have questions about the need to register error on the side of registration or consult the editorial office of the journal in which they wish to publish.

*US Public Law 110–85* (Food and Drug Administration Amendments Act of 2007 or FDAAA), Title VIII, Section 801 mandates that a “responsible party” (i.e., the sponsor or designated principal investigator) register and report results of certain “applicable clinical trials”: Trials of Drugs and Biologics: controlled, clinical investigations, other than Phase I investigations, of a product subject to FDA regulation; and Trials of Devices: controlled trials with health outcomes of a product subject to FDA regulation (other than small feasibility studies) and pediatric postmarket surveillance studies. NIH grantees must take specific *steps to ensure compliance* with NIH implementation of FDAAA.

### Confidentiality

Personal information about potential and enrolled participants will be collected and then de-identified prior to it being shared to ensure confidentiality of participants is maintained before, during, and after the study. Only the MPIs, the biostatistician, and other members of the research team will have access to the final study dataset.

### Access to Data

Only investigators and approved researchers added by ethics approval will have access to the final study dataset.

### Ancillary and Post-Study Care

The intervention has been developed by NYU Dentistry and a research team with expertise in oral health and health care. We believe that the need to discontinue the intervention will be extremely minimal. If any participant becomes distressed as a result of participation in our study, they will be referred to appropriate counseling support services.

### Dissemination Policy

Results from this feasibility and acceptability study will be provided to study participants and disseminated to oral health and health care professionals through presentation at seminars and conferences and publication in scientific journals and relevant media. We will adhere to all guidelines for authorship.

### Appendices

The patient consent brochure, non-patient consent brochure, and consent signature page will be provided to participants and their authorized surrogates and are available as Appendices.

## Strengths and Limitations

The strengths of this feasibility and acceptability study include the expertise and experience of the involved researchers, providers, and administrators, and the commitment of NYU Dentistry to improve its *Local Community Outreach Programs* to meet the needs of the local Chinese American community. Further, the *Center for the Study of Asian American Health* at NYU Medicine is the only center of its kind in the United States solely dedicated to research and evaluation on Asian American health and health disparities. Thus, this study holds the potential to fill a care gap for this diverse and growing population. In particular, Chinese are the largest Asian ethnic group in New York, NY, with higher poverty rates for working age and older adults relative to all residents (56). As NYU Dentistry and NYU Medicine have partnered on CBPR initiatives using CHW models (21, 29), there is confidence in our ability to adapt materials and programming for this new population/setting of Chinese American outreach sites. Further, in longstanding collaboration with The State University of New York at Buffalo, our research team has examined how factors at multiple levels contribute to oral health and care-seeking behaviors of racial/ethnic minority older adults (37, 38). Leveraging our portfolio of systems science models and group model-building expertise and experience, we plan to engage with our partners to understand the dynamic complexity of our interventions and simulate alternate scenarios, in concert with a recent NIH funding opportunity announcement (57). Finally, integrating multiple scientific approaches (implementation science, CBPR, and systems science) and utilizing remote EHR capabilities to enhance patient care and tracking are notable strengths of this study.

This feasibility and acceptability study also has certain limitations. First, findings may not be generalizable to other settings and locales. Nonetheless, by furnishing our protocol to the research community, other implementation scientists may adapt it for their local needs. Second, the study is only funded for 1 year. Thus, we will not be able to track patient participants screened during the second half of the funding period to determine whether or not they visited a dental provide in the complete follow-up year, as per Leading Health Indicator OH-7 (6). Still, this funding provides an opportunity to ascertain the feasibility and acceptability of our partnered intervention, and strengthen our methods toward designing and implementing a randomized controlled trial of a participatory, multi-level, partnered intervention to improve the oral and general health of low-income Chinese American adults. Further, while the pre-post retrospective evaluation design to assess the acceptability of the partnered intervention is considered a practical method to evaluate learning from an educational program (58), it has certain limitations. In particular, recall bias may affect the quality of the data collected due to potentially inaccurate or skewed memories of patients regarding their prior attitudes, emotions, behaviors, and experiences. Finally, social desirability bias may affect patients’ self-reported brushing of teeth for 2 min twice a day with a soft-bristled toothbrush and fluoride toothpaste.

## Ethics Statement

This feasibility and acceptability study will be conducted in accordance with the International Council on Harmonization guidelines for Good Clinical Practice (ICH E6), the Code of Federal Regulations on the Protection of Human Subjects (45 CFR Part 46), and the NIDCR Clinical Terms of Award. All subjects gave written informed consent in accordance with the Declaration of Helsinki. The protocol was approved by the IRBs at both NYU Langone Health (study i17-01077) and The State University of New York at Buffalo (study 1749).

## Author Contributions

MN conceived the study and led the writing. SM and QZ contributed systems science and geographic expertise. SY and CT-S contributed CBPR and evaluation expertise. XG contributed health policy and Chinese culture expertise. All authors contributed to the drafts and approved the final version of the paper.

## Conflict of Interest Statement

The authors declare that the research was conducted in the absence of any commercial or financial relationships that could be construed as a potential conflict of interest.

## Footnotes

^1^https://www.anylogic.com/.

^2^http://vensim.com/.
